# Army Veterinary Corps

**Published:** 1900-09

**Authors:** 


### EDITORIALS.

### ARMY VETERINARY CORPS.

(Concluded from page 508.)

The Chairman: Assuming that we must have trained veterinary surgeons—I doubt if there is very much difference of opinion on that point—the question is, how to get the best results from them. They are practically staff officers. It has been suggested by some very able men that the best results would be obtained by having them made regimental staff officers, except those who are in the Quartermaster's Department, who would be under the control of the quartermaster. That is the point, I think, upon which the committee would like to hear your views. This bill establishes a corps of veterinary surgeons, with a head, and the question is why the other system, by which they are subject to the commanding officers of the regiments and batteries, would not be as good or better than a veterinary corps which is subject to a central authority here. That is the question.

Mr. Marsh: That is the point of difference.

The Chairman: That is the point of difference upon which the committee wants information. There is no difference of opinion among the members of the committee, I assume, as to the desirableness and valuableness of veterinary surgeons in the Army.

Mr. Marsh: The question is whether they shall have a separate corps, such as is proposed in this bill, or whether they shall be a part and parcel of the Army itself, as a quartermaster, or an assistant surgeon, or regimental surgeon is on the regimental staff.

The Chairman: That is in the volunteer service.

Mr. Marsh: That is true. The proposition contained in this Senate bill involves the organization of an entirely new staff, the head of which is to be a colonel, and the head of which it will very soon be insisted should be a general, so that he may be on a plane with the heads of the eleven other staffs. That is the only question, I think, upon which we want to hear statements.

Colonel Huidekoper: To get absolute harmony of work the men, you know, must be supplied with the same instruments, the same books, and medical supplies, return uniform reports, be stationed where their ability can be used to the best advantage, etc. For that purpose I think a corps system is absolutely necessary. A man put in one place and kept there has his views confined, and consequently his views become narrow; if he is attached to a particular regiment, and sees no future of promotion into the better class, he does not get the wider experience which makes him a better officer. If a local man is assigned to a particular regiment and he happens to die, the regiment must wait until it is supplied, through another examination with another man who is tied right down to a particular class of work under a particular officer; whereas, under the other system, if a man happens to die, a first lieutenant of the Quartermaster's Department can be assigned to that regiment, and it will not have to wait three days until they will have a competent man assigned back to the duty until a future examination furnishes them with another man.

Mr. Marsh: That depends on whether you have some extra men on hand, not needed, as to whether you assign them within three days or not. If they are needed in some other position you would have to supply that other position as well as the first.

The Chairman: What is the rule in other countries in that regard? Are they staff officers or not?

Colonel Huidekoper: This book, published by the Adjutant-General's Office in January, 1899, gives the entire staff of every European army. Every staff organization has a veterinary surgeon attached to it—the French veterinary service, for instance, and, a little later, the one in England—with the exception of two countries, Roumania and Austro-Hungary. In those countries there is what is called a sanitary service, with one head, which includes three sub-departments—the medical officers, the apothecaries, and the veterinary surgeons. That condition existed in Belgium up until 1892, when the veterinary department was removed from the sanitary board and made a separate corps; but according to a letter which I received last autumn from the chief of the service, before 1892 the veterinary service was a part of the service of health of the army, which comprised the medical men, the pharmacists, and the veterinarians. The service was directed by a medical man, the inspector-general of the service of health of the army, having the rank of a major-general. The chief veterinarian was attached. Now the veterinary service is a separate department, reporting directly to the minister of war. Roumania, then, is the only one——

The Chairman: Colonel Marsh calls attention to the fact that the so-called Hull bill provided for a corps. It did not, as I understand.

Colonel Huidekoper: No, sir.

Mr. Marsh: I understood you to say that.

Colonel Huidekoper: No, sir. It provided for commissions for two officers to each cavalry regiment.

Mr. Marsh: I understood you to say that the provision of the Senate bill on this subject was substantially the same as that of the Hull bill of last Congress?

Colonel Huidekoper: No, sir; that was the amendment authorized by the President and the Secretary of War, which was offered as an amendment in the Senate. The Hull bill provided for two veterinarians, with the rank of second lieutenant.

The Chairman: That is what I understood you to mean.

Colonel Huidekoper: Then the Senate cut out that provision and gave the men the pay of second lieutenants and sergeants-major without rank.

Mr. Marsh: Did the Senate committee report that provision at the last Congress?

Colonel Huidekoper: No, sir; it came to the floor of the Senate with the indorsement of the Secretary of War. It was too late for the committee's action.

Mr. Marsh: Then it was not indorsed by the committee of this house, nor of the other, at the last Congress?

Colonel Huidekoper: No, sir.

Mr. Marsh: And this provision now in the Senate bill was not indorsed by the Committee on Military Affairs in the Senate, either?

Colonel Huidekoper: No, sir. It had the indorsement of General Miles, General Brooke, General Merritt, the commandant at West Point, the Quartermaster-General, and a large list of prominent army officers who had served for years.

Mr. Marsh: You say this system has their indorsement?

Colonel Huidekoper: Yes, sir.

Mr. Marsh: Have you got it there?

Colonel Huidekoper: I have copies of the letters of Generals Merritt, Brooke, and Wilson, Colonel Hein and others.

Mr. Marsh: Do they indorse the organization of a separate staff or bureau?

Colonel Huidekoper: All, with the exception of General Merritt. General Merritt objected to the formation of a new bureau, while at the same time his letter——

Mr. Marsh: Did General Miles favor the new bureau?

Colonel Huidekoper: Yes, sir.

Mr. Marsh: With a colonel at the head of it?

Colonel Huidekoper: Yes, sir.

Mr. Marsh: In what report does he favor it?

Colonel Huidekoper: General Miles?

Mr. Marsh: Yes; in what report does he so favor it?

Colonel Huidekoper: He has made no report, sir; it has never been submitted to him officially.

Mr. Marsh: He has never favored it officially?

Colonel Huidekoper: Not in an official indorsement; no, sir. It was never referred to him officially.

Mr. Marsh: Have these other generals favored it officially?

Colonel Huidekoper: No, sir; not in an official indorsement.

The Chairman: It was in writing to you?

Colonel Huidekoper: In writing to me.

The Chairman: They expressed their views in that way?

Colonel Huidekoper: Here are the originals. I have copies of them.

Mr. Marsh: Will you read them?

Colonel Huidekoper: Yes, sir, I will They are rather long, but I will read them if you wish.

The Chairman: If they are very long, you had better put them in the record.

Mr. Marsh: What is the date of the first one?

Colonel Huidekoper. General Merritt's letter is dated February 15, 1900.

Mr. Marsh: 1900?

Colonel Huidekoper: 1900. It is a letter addressed to me. I have his original letter here if you would like to see it.

Mr. Marsh: Just put them all in the record.

Colonel Huidekoper: They are all in the Congressional Record. They were printed there.

The Chairman: I think you will find them in the debates on the bill, but it is well enough to put them in here also.[1]

[1] These letters from Major-General Wesley Merritt, Major-General John R. Brooke, Major-General James H. Wilson, Brigadier-General J. C. Breckenridge, Brigadier-General Wallace F. Randolph, Brigadier-General Charles King, General James A. Beaver (Ex-Governor of

Colonel Huidekoper: Since that time General Brooke has come to Washington, and I have consulted him personally in regard to this bill, and have his approval of it.

Mr. Marsh: In regard to what bill?

Colonel Huidekoper: In regard to section 14 of this bill, the amendment offered by Senator Kenney in the Senate.

The Chairman: Was it in existence at that time?

Colonel Huidekoper: Oh, yes, sir; Senator Kenney gave notice of the proposed amendment on the 2d of March.

The Chairman: But this letter was written in December?

Colonel Huidekoper: I have just stated that since that letter was written General Brooke has been here, up to a week or so ago, and I consulted him personally in regard to it.

Mr. Marsh: Practically, the question here, Colonel (without consuming more time than is necessary), is whether this veterinarian force shall be a part of the Army, or whether it shall be a separate staff organization?

The Chairman: That is the whole question.

Mr. Marsh: Everybody understands the importance of veterinary surgeons.

Colonel Huidekoper: I think that, to get effective work, an independent organization is necessary, the head of which, while the men are directly subservient to the officer to whom they are assigned for work, will be a channel through which information can be obtained as to the class of work they do, so that the results can be compared and their efficiency known. An officer who is very efficient under one commanding officer might not be under another. He might become excessively lax under one commanding officer, and yet be an efficient man if placed at work somewhere else, with different surroundings.

Mr. Marsh: Of course, in the practical operation of this system, you would have to place a veterinary surgeon where he could get along harmoniously and develop his best ability?

Colonel Huidekoper: Yes, sir.

Mr. Marsh: And instead of making him conform to conditions you would have conditions conform to him?

Colonel Huidekoper: On the contrary, I would make him conform to the conditions.

Pennsylvania), Colonel O. L. Hein (Commandant of Cadets, West Point), Colonel J. G. C. Lee (Asst. Q. M. General), Colonel D. D. Wheeler (Deputy Q. M. General), Major Jno. W. Pullman (late Chief Q. M. Puerto Rico) have been published in pamphlet form and in the Congressional Record, May 4, 1900.

Mr. Marsh: Well, you would not be doing that if you changed him from place to place. You would be hunting for a condition of things which would suit him, instead of making him suit the condition of things at the place where he was ordered to report.

Colonel Huidekoper: I think there are plenty of means in the Army, if a man is not efficient, and does not do what his duties require him to do, of getting rid of him.

Mr. Marsh: But you said he might get to be very efficient under one officer and not be efficient under another, and consequently you would change him, so that he would not have to adapt himself to conditions.

Colonel Huidekoper: I have seen this same thing occur in the Medical Department, where a commanding officer preferred not to have a particular medical officer assigned to him for any great length of time, but preferred to have another medical officer sent to him.

Mr. Marsh: I have no doubt that exists.

The Chairman: I see that France has two generals in the veterinary department.

Colonel Huidekoper: The forces in France and Germany have been increased 40 per cent. since this book was published.

Mr. Capron: I have been trying, Doctor, as you have gone along, to see if I could get into my mind the reasons which you give for the establishment of a separate bureau; and I have put them down in the way that I will read to you. See if I understand you correctly: "The examination of applicants for admission and for promotion; the inspection of animals at time of purchase, and periodically afterward; the prevention of disease; the proper supply of medicines and implements, and a bureau for the promulgation of new theories in regard to practice as they may develop in further and better knowledge." Is that correct?

Colonel Huidekoper: Yes, sir.

Mr. Capron: Those things would result from the establishment of a bureau, and would never come——

Colonel Huidekoper: They would never come from the simple employment of individuals any more than in building a house, where you require different classes of work for mortar work and carpentry, you cannot get along without a foreman to see that everything works harmoniously.

Mr. Capron: Or an architect to inspect the whole thing after the house is finished?

Colonel Huidekoper: To see that it is properly done; yes.

Mr. Lentz: It is your idea, Doctor, that a man, when he gets into a corps, or into the service, should remain there for life? You speak of having a bureau, and having an organization so as to determine the efficiency of the different men. Now, suppose a man proves a failure after he has passed an examination and been admitted?

Colonel Huidekoper: If he does not do his duty, he can be gotten rid of.

Mr. Lentz: How?

Colonel Huidekoper: By court-martial, on charges of inefficiency. As you will see, this bill provides for a series of four examinations, and a more thorough course of examination for promotion than exists in any other department. The Medical Department has but one, and then a final one for majority. In this case, each promotion is to be (if you will look at the wording of the bill) a "satisfactory competitive examination," to pick out the best men for each promotion.

Mr. Lentz: Satisfactory to whom; simply to the Secretary of War?

Colonel Huidekoper: To the board of examiners.

Mr. Marsh: Who compose that board of examiners?

Colonel Huidekoper: That board will be appointed by the Secretary of War, sir. The present board is made up of two cavalry officers—that is, under the old law—and a veterinary surgeon from the Department of Agriculture. For the elementary medical studies, I should say that a surgeon from the Medical Department should be on the board, for examination in such things as bacteriology, the use of the microscope, chemistry, physiology, and the principles of medicine, which are common to both branches.

Mr. Lentz: Is it not a fact, Doctor, that the tabulating of the results of this kind of work would probably be more serviceable to the farmers than that which is now done by the Agricultural Department?

Colonel Huidekoper: It would be the only means of getting information on this subject, so as to enable us to economize and save money. To-day, when the Quartermaster-General sends an enormous quantity of supplies out to Manila on the requisition of a quartermaster the requisition is unquestionably written in the first instance by a veterinary surgeon who does not belong to the Army, and who cannot officially sign a requisition. The veterinary surgeon in Manila writes out an immense list, on which the names of twenty drugs are misspelled, and the quartermaster signs that requisition. This large quantity of drugs is sent back to Manila, and there is no record of what animals are sick, what they die of, or of the utility of sending these supplies out there, which information such a service as this would give.

Mr. Marsh: That is due to the want of competent veterinarians in the Army at the present time, is it?

Colonel Huidekoper: Yes; and to the want of system. They are civilians. They cannot sign requisitions. The quartermaster has to do it.

If you will allow me to cite just one case, I will say that when the regular cavalry went to Chickamauga in the spring of 1898, some three veterinary surgeons went there with them. Only one had any instruments of any kind whatever, because the other two had left regimental quartermasters who were responsible for those instruments, and would not let them go out of the post from which they came. The regimental quartermaster, in such case, is the one who makes requisition for the instruments, and loans them to the veterinary surgeon each time he wants to use them. The veterinary surgeon is not responsible for them; and these two veterinary surgeons had to go to the third one to get scissors and needle and thread to stitch up the wounds in the horses at Chickamauga.

Mr. Marsh: All that could be remedied without an independent staff corps, however, could it not?

Colonel Huidekoper: I myself do not see how an efficient organization could be obtained in any other way.

Mr. Lentz: What is the objection to a staff corps? If we were going to use the same number of men and the same amount of service, why not give them a separate organization and make them responsible?

The Chairman: Colonel Huidekoper's theory is that you should.

Colonel Huidekoper: I cannot see any other way of getting an effective organization.

Mr. Lentz: If we are going to have the same number of men in any event, I cannot see why that would not be the best plan.

Colonel Huidekoper: It seems to me that in doing that we would only be following the precedent set by every European country. As an example: Belgium, within the last seven years (in 1892), removed her veterinarians from the general sanitary department and made them a separate corps, because she found it necessary, as had also her neighbors, France and Germany.

Mr. Lentz: Doctor, you speak of a requisition being made for medicines or remedies from Manila. Did you see or examine the requisition yourself?

Colonel Huidekoper: Yes, sir; I went over it. I know the officers of the Quartermaster's Department very well; and just as a personal matter I was asked to go over this requisition to see what was necessary, and to see if it was a satisfactory requisition.

Mr. Lentz: That was what you were asked by whom?

Colonel Huidekoper: By the Quartermaster-General.

Mr. Lentz: Now, in your examination of that requisition, did it meet with your approval as to the proper proportions of the remedies?

Colonel Huidekoper: No; it was a requisition that contained proprietary medicines, such as "Listerine," that costs seventy-five cents a bottle, for the use of horses—a medicine that could be made up with the crude drug and sent out in a more compact form, and which, with the addition of so much fresh water and a bit of boracic acid, eucalyptol, or something of the kind, would make just as effective an article and answer the purpose just as well at a cost of a cent a bottle instead of seventy-five cents.

I recollect that same requisition contained numerous other drugs and proprietary medicines, or medicines in bottles, requiring to be sent from here to Manila in a very bulky form, which could have been sent in the crude form just as well. Take, for instance, tincture of iodine, put up in 8-ounce bottles. If we sent them crude iodine in a box, they could have made their own tincture of iodine (as alcohol is easily obtainable in Manila), instead of having it transferred in bottles, if the men had the proper outfit of instruments, materials, and supplies for the purpose. But when you compare the list of veterinary instruments, books, and medicines furnished the United States Army, which covers less than a page, with the complete supplies for a troop of cavalry, for a regiment, for the officers, and for headquarters, as given in the French regulations, you see that one is thoroughly systematic and the other is not.[1]

Mr. Lentz: Do you mean to say that, by reason of the want of system, we are now guilty of extravagance?

Colonel Huidekoper: Under the present system we are guilty of enormous extravagance, not only in the amount of medicine used, but in the loss of animals, for which there is no necessity. As medical officer of the First Army Corps I took to Porto Rico, under my own direction, 180 hospital animals. I had no authority to employ veterinary services. I fortunately found that I had in my hospital corps an enlisted man, who was a veterinary surgeon, from St. Louis—a very clever fellow, as he proved—and I made him an acting steward and put him in the ambulance company. He came to me for certain things, which I purchased out of my own pocket, but we could not get anything in large quantities, and I had to do what everybody else did. When animals were ailing I went to the quartermaster and drew so many good ones, and I turned back to him animals which with the slightest attention would have kept well, but which without the possibility of attention immediately got large sores and grew worse and worse. The easiest way to do, as everyone did, was to turn them over to the quartermaster and get fresh ones and let him take the responsibility of having them go to pieces.

In the case of the batteries at Coamo—Major Lancaster's battery—I happened to know all the officers personally; and I was making a medical inspection one day and they asked me if I would not give them half an hour for the purpose of looking at their horses. I did so, and as a result of that examination a captain went into the town and purchased out of his own pocket a few simple things for taking care of the sores of these horses, for which there had been no provision made, and which the farrier of the battery ought to have had (and in every other army would have had) right in the caisson of one of the guns, or in what is known as the veterinary caisson, which goes with every battery.

Mr. Lentz: What proportion or percentage of the requisition of which you spoke, made for Manila, for instance, could have been saved through judicious distribution of the packages and judicious selection of the articles, in accordance with your

[1] Reference was here made to copies of the detailed regulations of the Veterinary Service in the French, Belgian and other armies, which were shown in evidence.

suggestion, instead of sending them in the form they were asked for?

Colonel Huidekoper: I recollect that out of a long three-page requisition (written on three pages of legal cap, with each line filled) there were some dozen or fifteen articles that were absolutely unnecessary, and a half a dozen or a dozen more that could have been put up in less expensive form than that called for, and in a form which would have been easier to handle. On the other hand, there were certain essential things that were left out.

